# Residential Road Traffic Noise and High Depressive Symptoms after Five Years of Follow-up: Results from the Heinz Nixdorf Recall Study

**DOI:** 10.1289/ehp.1409400

**Published:** 2015-11-25

**Authors:** Ester Orban, Kelsey McDonald, Robynne Sutcliffe, Barbara Hoffmann, Kateryna B. Fuks, Nico Dragano, Anja Viehmann, Raimund Erbel, Karl-Heinz Jöckel, Noreen Pundt, Susanne Moebus

**Affiliations:** 1 Centre for Urban Epidemiology (CUE), Institute for Medical Informatics, Biometry and Epidemiology (IMIBE), University Hospital Essen, Essen, Germany; 2 IUF-Leibniz Research Institute for Environmental Medicine, Düsseldorf, Germany; 3 Medical School, Deanery of Medicine, the Heinrich Heine University of Düsseldorf, Düsseldorf, Germany; 4 Institute for Medical Sociology, Centre for Health and Society, Medical Faculty, University of Düsseldorf, Düsseldorf, Germany; 5 IMIBE, University Hospital Essen, Essen, Germany; 6 Department of Cardiology, University Hospital Essen, Essen, Germany

## Abstract

**Background:**

Traffic noise affects a large number of people, particularly in urbanized areas. Noise causes stress and annoyance, but less is known about the relationship between noise and depression.

**Objective:**

We investigated the association of residential road traffic noise with depressive symptoms using 5-year follow-up data from a German population-based study.

**Methods:**

We analyzed data from 3,300 participants in the Heinz Nixdorf Recall study who were between 45 and 75 years old and were without depressive symptoms at baseline (2000–2003). Depressive symptoms were defined based on the Center for Epidemiologic Studies Depression scale (CES-D) 15-item questionnaire (total score ≥ 17) and antidepressant medication intake. Road traffic noise was modeled according to European Parliament/Council Directive 2002/49/EC. High noise exposure was defined as annual mean 24-hr noise levels > 55 A-weighted decibels [dB(A)]. Poisson regression with robust variance was used to estimate relative risks (RRs) a) adjusting for the potential confounders age, sex, socioeconomic status (SES), neighborhood-level SES, and traffic proximity; b) additionally adjusting for body mass index and smoking; and c) additionally adjusting for the potential confounders/intermediates comorbidities and insomnia.

**Results:**

Overall, 35.7% of the participants were exposed to high residential road traffic noise levels. At follow-up (mean = 5.1 years after baseline), 302 participants were classified as having high depressive symptoms, corresponding to an adjusted RR of 1.29 (95% CI: 1.03, 1.62; Model 1) for exposure to > 55 versus ≤ 55 dB(A). Adjustment for potential confounders/intermediates did not substantially alter the results. Associations were stronger among those who reported insomnia at baseline (RR = 1.62; 95% CI: 1.10, 2.59 vs. RR = 1.21; 95% CI: 0.94, 1.57) and appeared to be limited to those with ≤ 13 years of education (RR = 1.43; 95% CI: 1.10, 1.85 vs. 0.92; 95% CI: 0.56, 1.53 for > 13 years).

**Conclusion:**

Our results suggest that exposure to residential road traffic noise increases the risk of depressive symptoms.

**Citation:**

Orban E, McDonald K, Sutcliffe R, Hoffmann B, Fuks KB, Dragano N, Viehmann A, Erbel R, Jöckel KH, Pundt N, Moebus S. 2016. Residential road traffic noise and high depressive symptoms after five years of follow-up: results from the Heinz Nixdorf Recall Study. Environ Health Perspect 124:578–585; http://dx.doi.org/10.1289/ehp.1409400

## Introduction

Noise is a psychosocial stressor that may affect health, even at low levels (Babisch 2002). A large number of people in urban settings are exposed to traffic noise, and the World Health Organization (WHO) considers environmental noise to be an important public health issue (WHO 2011). Beyond causing annoyance, exposure to traffic noise has been associated with stress-related and cardiovascular outcomes such as hypertension and myocardial infarction (Barregard et al. 2009; Fuks et al. 2011; Willich et al. 2005). Recently, an association of long-term exposure to traffic noise with incident diabetes mellitus type 2 has been reported (Sørensen et al. 2013). Until now, epidemiologic research on noise has focused mainly on cardiovascular effects, but less is known about the relationship between traffic noise and mental health problems such as depression.

Depression is a common mental disorder and an increasing public health concern (Weissman et al. 1992), and it is a leading cause of disability worldwide. According to results reported in the Global Burden of Diseases, Injuries, and Risk Factors Study 2010, mental and substance use disorders contributed 7.4% to the total global burden of disease [as measured in disability-adjusted life years (DALYs)] in 2010, of which 40.5% was attributable to depressive disorders (Whiteford et al. 2013). Individuals affected by depression not only experience reduced quality of life due to suffering but also may be unable to cope with everyday life tasks including performing occupational activities, which results in increased sick leave (Wedegaertner et al. 2013).

The etiology of depression is multi-factorial and complex. Psychological, social, and biological factors may be involved, most likely in combination (WHO 2012). The potential influence of noise on mental health has been examined, but findings from studies of noise and mental health outcomes have been inconsistent (Crombie et al. 2011; Floud et al. 2011; Hardoy et al. 2005; Niemann et al. 2006; Schreckenberg et al. 2010; Sygna et al. 2014). These discrepancies may be attributed to differences in study design, investigated populations (children, adults), exposures (aircraft and road traffic noise and subjective noise annoyance as opposed to objectively modeled/measured noise), and outcomes (various psychological symptom measures/questionnaires, diagnoses, medication intake, mental hospital admissions). Few studies have examined the association between road traffic noise and depressive symptoms in adults, and there is a particular lack of evidence from prospective studies. To our knowledge, there is only one prospective study that has examined this association (Stansfeld et al. 1996). This study was conducted in Caerphilly, South Wales, and the authors found no association between traffic noise levels at baseline and depression scores after 5 years of follow-up; however, only men (*n* = 1,725) were included.

There are several proposed pathways supporting the hypothesis that chronic noise exposure may be related to depressive symptoms. Sleep disturbance conditions such as insomnia, which may be caused by traffic noise (Halonen et al. 2012), have been shown to be associated with depression in previous studies (Franzen and Buysse 2008; Riemann and Voderholzer 2003; Roberts et al. 2000). Thus, decreased quality of sleep represents one possible link between noise exposure and mental health. A recent cross-sectional study analyzing survey data for 2,778 adults from an age- and sex-stratified population registry sample in Oslo, Norway, found a weak association between road traffic noise and mental health as measured by the Hopkins Symptom Checklist, but only in participants with poor quality of sleep (Sygna et al. 2014). Furthermore, acute noise events cause biological stress reactions (Babisch 2002). Such stress reactions may in turn promote onset of depression (Anisman and Merali 2002; Wager-Smith and Markou 2011); however, single acute noise events are unlikely to cause depression. Thus, the question whether repeated or chronic noise exposure has long-term effects on depressive illness is unresolved.

The aim of this study was to investigate the association of long-term exposure to objectively measured road traffic noise with depressive symptoms within a population-based cohort of middle-aged men and women living in the highly urbanized metropolitan Ruhr area in Germany.

## Methods

**Figure 1:**
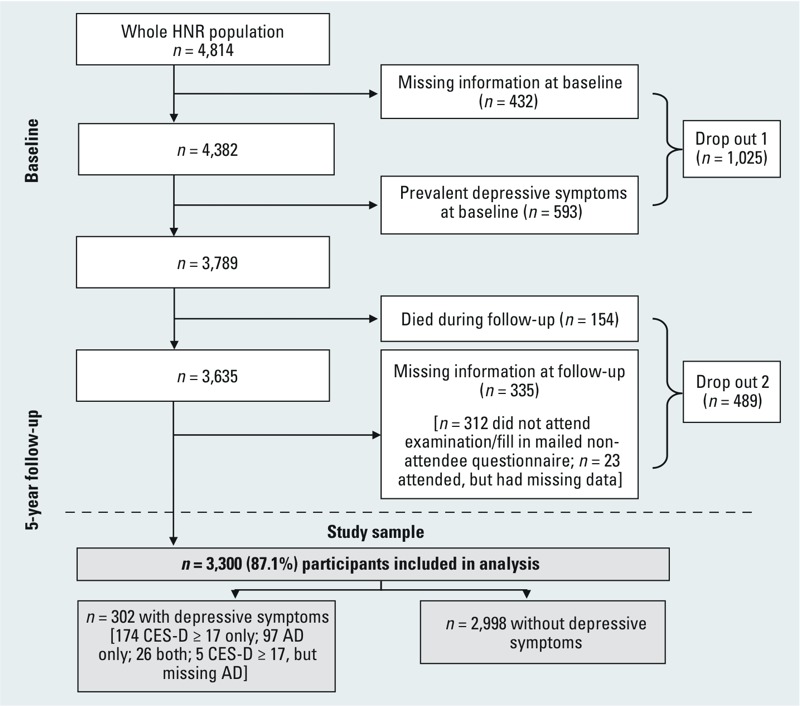
Flow chart of study participants in the Heinz Nixdorf Recall (HNR) study. Missing information = missing information on depressive symptoms [Center for Epidemiologic Studies Depression Scale (CES-D), antidepressant medication use (AD)]; prevalent depressive symptoms = CES-D ≥ 17 and/or antidepressant medication use.

*Study population.* We analyzed baseline and 5-year follow-up data from the ongoing prospective Heinz Nixdorf Recall study (HNR) conducted in three large adjacent cities (Bochum, Essen, and Mülheim/Ruhr) located in western Germany. The study design has been described in detail elsewhere (Schmermund et al. 2002). Baseline examinations were performed between 2000 and 2003 and included 4,814 participants between 45 and 75 years old who were randomly selected from population registries. Individuals were eligible if their address was valid, they were not institutionalized, had sufficient knowledge of the German language, were not severely ill, and were able to be interviewed. In addition, pregnant women (although not a priority, given the investigated age group) and relatives of study personnel were excluded. The baseline response calculated as recruitment efficacy proportion was 55.8% (Stang et al. 2005). Follow-up examinations were performed between 2005 and 2008. Our analyzed sample is depicted in Figure 1 and is further described in the statistical analysis section of the “Methods.” The study maintains extensive quality management procedures, including a certification according to Deutsches Institut für Normung (DIN) ISO 9001:2000/2008 (DIN 2000). The HNR was approved by the local ethics committees, and all participants gave informed consent prior to participation.

*Outcome.* Depressive symptoms during the previous week were assessed using the 15-item short-form questionnaire of the Center for Epidemiologic Studies Depression Scale (CES-D) (Hautzinger and Bailer 1993; Radloff 1977), which was distributed to participants at the baseline and 5-year follow-up visits at the study center (and was mailed to participants who did not attend the examinations). The CES-D is a screening tool for measuring depressive symptoms; it has been validated in different populations and settings and is frequently used in health research (Radloff 1977). Possible scores for the 15-item version range from 0 to 45, with higher levels indicating more and/or more frequent depressive symptoms. The CES-D is considered an indicator of a probable depressive episode but does not replace a face-to-face physician diagnosis. Antidepressant medication was also included in the outcome definition because it is indicative of depressive symptoms being treated (even if off-label use may occur) and may affect CES-D results in depressive individuals because treated participants may show fewer symptoms of depression. Assessment of all medication intake was performed by asking participants to bring all medication (including packages) taken in the previous 7 days to both the baseline and follow-up visits. Intake of antidepressant medication classified in the Anatomical Therapeutic Chemical (ATC) groups N06A or N06CA [WHO Collaboration Centre for Drug Statistics Methodology (WHOCC) 2011] and/or a CES-D score ≥ 17 according to Hautzinger and Bailer (1993) were used to define high depressive symptoms.

*Exposure.* Road traffic noise was modeled according to Directive 2002/49/EC of the European Parliament and Council of the European Union (2002) for the year 2006 as a weighted day–evening–night (24-hr) average sound level (L_den_) in 5–A-weighted decibel [dB(A)] categories (isophones). The following factors were considered in the noise-level modeling: small-scale topography of the area, dimensions of buildings, noise barriers, street axis, vehicle type–specific traffic density, speed limit, and type of road surface. Noise exposure data were assigned to the geographic residence location of the study participant at baseline using the geographic information system ArcGIS, assuming average noise levels to be relatively stable over time. High noise exposure was defined as noise levels of L_den_ > 55 dB(A), based on the maximum community noise levels recommended by the WHO (Berglund et al. 1999). Data on nighttime noise (L_night_, 2200–0600 hours) were available and were also analyzed, with nighttime noise levels > 50 db(A) defined as high noise exposure.

*Covariates.* Socioeconomic (e.g., income), demographic (e.g., age), behavioral (e.g., smoking: current, former, or never smoker), and medical history data were assessed via standardized computer-assisted personal interviews at the baseline examination. Education, income, and economic activity were used as indicators of socioeconomic status (SES) (Shavers 2007; Galobardes et al. 2007). Education was defined by combining school and vocational training as total years of formal education, according to the International Standard Classification of Education (UNESCO 1997), and was categorized into four groups (≤ 10, 11–13, 14–17, and ≥ 18 years). Income was measured as the monthly household equivalent income, which was calculated by dividing the total household net income by a weighting factor for each household member, and was divided into four groups using sex-specific quartiles. Economic activity was categorized into three groups [employed, inactive (retired, homemaker, etc., but not unemployed), and unemployed]. Information on whether participants had/had ever had myocardial infarction, heart failure, stroke, diabetes mellitus, emphysema, asthma, cancer, rheumatism, slipped disc, or migraine (yes/no) at baseline was used to create a categorical variable indicating the number of comorbidities (0, 1, or ≥ 2). In addition, participants were asked to indicate if they had/had ever had depression. Insomnia was assessed based on three insomnia symptoms: difficulties falling asleep, difficulties maintaining asleep, and early morning arousals (Riedel et al. 2012). If participants reported that all of these symptoms were present at least two times per week during the previous 4 weeks, they were classified as having insomnia. One example of the three insomnia questions is “How often, during the last 4 weeks, did you have difficulties in falling asleep?” The possible answers were “never,” “sometimes (one time per week or less),” “often (at least 2 times per week),” or “almost every night.” Height and weight were obtained from standardized anthropogenic measurements performed during the clinical examination. The body mass index (BMI) was calculated as [weight in kilograms/(height in meters)^2^].

We applied the 2001 unemployment rate in the respective city unit (German terms: in Essen, “Stadtteil”; in Bochum and Mülheim/Ruhr, “Statistischer Bezirk”) as an indicator of neighborhood-level SES. These data were obtained from the local census authorities of the respective cities of Bochum, Essen, and Mülheim/Ruhr.

Residential distance to the nearest major road was calculated as a marker of traffic proximity using ArcGIS. A major road was defined as one falling into the upper quartile of mean daily traffic density (> 22,980 vehicles per day, year 2000). There was a weak negative correlation between traffic proximity and noise in our study (Pearson *r* = –0.22). We included this variable in the analysis to control for nonacoustic factors of traffic and the physical environment of the neighborhood (e.g., aesthetic aspects and perceived safety) that might affect mental wellbeing.

*Statistical analyses.* From the full HNR sample (*n* = 4,814), we excluded 432 participants with missing information on depressive symptoms (CES-D and/or antidepressant medication) and an additional 593 participants with prevalent high depressive symptoms at baseline (Figure 1). Of the remaining 3,789 participants, 154 died during follow-up, 312 were excluded because they did not attend the follow-up examination (when medication use and CES-D were assessed) or complete the mailed nonattendee follow-up questionnaire (including the CES-D), and 23 were excluded because they did not complete the CES-D and were not identified as using antidepressant medication at the follow-up visit (Figure 1). Five of the included participants did not attend the follow-up visit but were classified as having high depressive symptoms based on the mailed nonattendee follow-up CES-D. Thus, the final analysis sample included 3,300 participants (87.1% of the 3,789 eligible participants).

We used Poisson regression with a robust variance to estimate crude and adjusted effects of high road traffic noise on depressive symptoms after 5 years (Spiegelman and Hertzmark 2005; Zou 2004). The adjustment sets were selected *a priori* based on a directed acyclic graph (see Supplemental Material, Figure S1) created with DAGitty (Textor et al. 2011). In model 1, we adjusted for age (continuous), sex, education (four categories), income (quartiles), economic activity (three categories), neighborhood-level SES (unemployment rate, continuous) and traffic proximity (continuous). In Model 2, we additionally adjusted for the potential confounders BMI (continuous) and smoking, and in Model 3, the potential confounders/intermediates comorbidities (0, 1, or ≥ 2) and insomnia (yes/no) were added. Observations with any missing covariate data were automatically excluded from the respective analysis (complete case analysis). All analyses were also stratified by sex to investigate potential sex-specific differences. In addition to modeling road traffic noise as a binary variable [L_den_ > 55 vs. ≤ 55 dB(A)], we estimated associations with three noise exposure categories [L_den_ > 55 to ≤ 60 dB(A), > 60 to ≤ 65 dB(A), > 65 dB(A)] compared with the reference group that had L_den_ ≤ 55 dB(A) noise exposure.

We conducted exploratory analyses by stratifying the participants by *a*) education level (≤ 13 vs. > 13 years of formal education), *b*) movers versus nonmovers between the baseline and 5-year follow-up visits, *c*) insomnia (yes/no), and *d*) city of residence. Further sensitivity analyses were conducted by *e*) additionally excluding participants who reported to have/ever have had depression at baseline, *f*) using a cutoff of L_den_ > 65 dB(A) to define very high noise exposure, *g*) using CES-D score ≥ 17 exclusively to define high depressive symptoms at baseline and follow-up, and *h*) using antidepressant medication intake exclusively to define high depressive symptoms at baseline and at follow-up.

All analyses were conducted with SAS v.9.4 (SAS Institute Inc.).

## Results

Baseline characteristics of the analyzed population by noise exposure are shown in Table 1. Participants with high and low noise exposure were similar regarding sex and mean age, whereas proportions of insomnia, low education, low income, unemployment, and active smoking were higher in participants exposed to high noise levels. Only a small amount of covariate data were missing (maximum 15, for insomnia), with the exception of the income variable, for which a total of 196 values were missing (Table 1). Additionally, 605 values were missing for the variable indicating reported (lifetime) prevalence of depression, which was applied in one of the sensitivity analyses. At follow-up (5.1 years after baseline, on average), 302 participants [9.2%, including 201/1,585 women (12.7%) and 101/1,715 men (5.9%)] were classified as having high depressive symptoms based on a CES-D score ≥ 17 (*n* = 179), use of antidepressant medication (*n* = 97), or both (*n* = 26) in the previous week (Figure 1). Participants who were excluded from the analysis because of depressive symptoms/missing depressive symptoms data at baseline (drop out 1), or death or missing outcome data at follow-up (drop out 2), were similar to the analysis sample with regard to sex, age, and other baseline characteristics (see Supplemental Material, Table S1). However, they were more likely to have been current smokers (26–31% vs. 20–24%), and they had more comorbidities (36–37% vs. 29–31% with ≥ 2), lower education (19% vs. 8–9% with ≤ 10 years), and lower income (33–34% vs. 21–27% in the lowest quartile) than participants who were included in the analysis. Participants excluded because of prevalent depressive symptoms at baseline/missing depressive symptoms data were more likely to have reported insomnia at baseline (22% vs. 8–11%) and were less likely to be male (40% vs. 52%) than those who were included.

**Table 1:** Characteristics of the analyzed Heinz Nixdorf Recall study population (*n *= 3,300), by 24-hr road traffic noise.

Characteristic	L_den_ > 55 dB(A)	L_den_ ≤ 55 dB(A)
	*n* (percent), mean ± SD, or median (Q1, Q3)	*n* (percent), mean ± SD, or median (Q1, Q3)
Baseline
*n* (percent)	1,179 (35.7)	2,121 (64.3)
Men	610 (51.7)	1,105 (52.1)
Age (years)	59.1 ± 7.7	59.3 ± 7.6
Insomnia	124 (10.5)	177 (8.4)
Missing (*n*)	3	12
Number of comorbidities^*a*^
0	440 (37.3)	830 (39.1)
1	374 (31.7)	687 (32.4)
≥ 2	365 (31.0)	604 (28.5)
Reported (lifetime) prevalence of depression	70 (7.3)	106 (6.1)
Missing (*n*)	225	380
Body mass index	27.9 ± 4.7	27.7 ± 4.5
Missing (*n*)	6	4
Smoking
Current	288 (24.4)	423 (19.9)
Former	419 (35.5)	778 (36.7)
Never	472 (40.0)	920 (43.4)
Distance to nearest major road (meters)	532.4 (220.0,1083.1)	987.7 (552.8,1620.7)
Missing (*n*)	0	5
Unemployed in neighborhood (percent)	12.8 ± 3.3	12.0 ± 3.3
Education (years)^*b*^
≤ 10	111 (9.4)	165 (7.8)
11–13	703 (59.6)	1,135 (53.5)
14–17	251 (21.3)	525 (24.8)
≥ 18	114 (9.7)	295 (13.9)
Missing (*n*)	0	1
Household net income
Quartile 1 (low)	300 (27.0)	420 (21.1)
Quartile 2	257 (23.1)	473 (23.8)
Quartile 3	290 (26.1)	502 (25.2)
Quartile 4 (high)	266 (23.9)	596 (29.9)
Missing (*n*)	66	130
Economic activity
Employed	503 (42.7)	937 (44.2)
Inactive	591 (50.2)	1,078 (50.8)
Unemployed	84 (7.1)	106 (5.0)
Missing (*n*)	1	0
City of residence
Mülheim/Ruhr	467 (39.6)	772 (36.4)
Bochum	334 (28.3)	654 (30.8)
Essen	378 (32.1)	695 (32.8)
Follow-up
CES-D ≥ 17 and/or antidepressant medication	127 (10.8)	175 (8.3)
CES-D ≥ 17	89 (7.6)	116 (5.5)
Antidepressant medication	56 (4.8)	67 (3.2)
Missing (*n*)^*c*^	2	3
Moved between baseline and follow-up
Yes	214 (18.2)	314 (14.8)
No	965 (81.9)	1,807 (85.2)
Abbreviations: CES-D, Center for Epidemiologic Studies Depression Scale; dB(A), A-weighted decibels; L_den_, average annual 24-hour noise level; Q1, quartile 1 (25th percentile); Q3, quartile 3 (75th percentile). ^***a***^Of the following: myocardial infarction, heart failure, stroke, diabetes, emphysema, asthma, cancer, rheumatism, slipped disc, migraine. ^***b***^Combines school and vocational training. ^***c***^These participants were identified as having high depressive symptoms by CES-D and were therefore included.		

Of the included study population, 35.7% (*n* = 1,179) were exposed to high 24-hr traffic noise levels [L_den_ > 55 dB(A)], and 25.8% (*n* = 850) were exposed to high traffic noise at night [L_night_ > 50 dB(A)]. Distributions of annual mean noise exposures (overall and at night) were positively skewed (see Supplemental Material, Figure S2).

The results of the regression analysis (Table 2) revealed an adjusted RR (Model 1) of 1.29 (95% CI: 1.03, 1.62) for high depressive symptoms at follow-up in participants exposed to high noise levels compared with the low-noise exposure group. Estimates for men and women combined were similar for Models 2 and 3 and the unadjusted estimate (Table 2). Unadjusted associations were stronger for men than for women but were similar between men and women after adjustment for sociodemographic covariates (Model 1) and BMI and smoking (Model 2). Adjusting for potential intermediates (comorbidities and insomnia, Model 3) slightly reduced the RR toward the null for men but did not influence the association for women. We excluded participants with missing income data (*n* = 196), which produced no substantial influence on the results, yielding a crude total RR of 1.39 (95% CI: 1.11, 1.74; *n* = 3,104) and an RR of 1.43 (95% CI: 0.97, 2.10; *n* = 1,652) in men and an RR of 1.36 (95% CI: 1.03, 1.78; *n* = 1,452) in women (data not shown in Table 2). In general, associations between depression and exposure to noise at night [L_night_ > 50 vs. ≤ 50 dB(A)] were similar to associations with average 24-hr noise exposure (Model 1 RR = 1.29; 95% CI: 1.01, 1.64 for men and women combined), although associations were weaker for men (RR = 1.19; 95% CI: 0.77, 1.82) than for women (RR = 1.36; 95% CI: 1.01, 1.82) (see Supplemental Material, Table S2).

**Table 2:** Relative risks (with 95% confidence intervals) of high depressive symptoms at follow-up in study participants exposed to residential road traffic noise (L_den_) > 55 dB(A) and L_den _≤ 55 dB(A).

Model	Cases (*n*)	Total (*n*)^*a*^	RR (95% CI)
Unadjusted
Total	302	3,300	1.31 (1.05, 1.62)
Men	101	1,715	1.46 (1.00, 2.13)
Women	201	1,585	1.23 (0.95, 1.60)
Model 1^*b*^
Total	279	3,098	1.29 (1.03, 1.62)
Men	98	1,650	1.29 (0.87, 1.92)
Women	181	1,448	1.30 (0.98, 1.72)
Model 2^*c*^
Total	278	3,089	1.28 (1.02, 1.61)
Men	98	1,644	1.28 (0.85, 1.94)
Women	180	1,445	1.28 (0.97, 1.69)
Model 3^*d*^
Total	276	3,075	1.26 (1.00, 1.58)
Men	97	1,637	1.21 (0.81, 1.82)
Women	179	1,438	1.28 (0.97, 1.70)
Abbreviations: CI, confidence interval; dB(A), A-weighted decibels; RR, relative risk. ^***a***^Numbers in Models 1-3 differing from the unadjusted model reflect missing covariate data. ^***b***^Adjusted for age, sex (except in the sex-stratified analysis), education, income, economic activity, neighborhood-level socioeconomic status, traffic proximity. ^***c***^Additionally adjusted for body mass index, smoking. ^***d***^Additionally adjusted for comorbidities, insomnia.			

**Figure 2:**
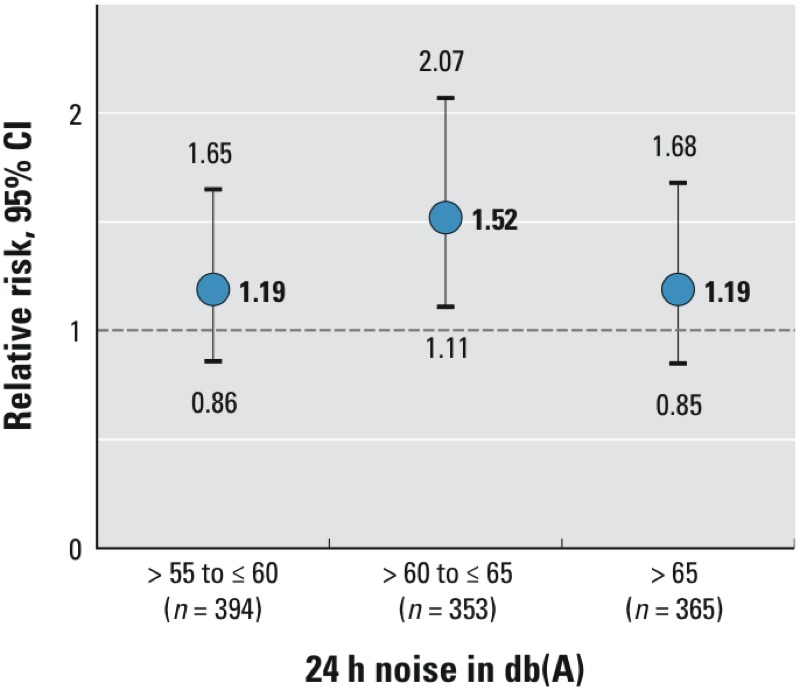
Relative risks and 95% confidence intervals of high depressive symptoms at follow-up in association with exposure to different categories of 24-hr noise compared with the lowest noise category [≤ 55 dB(A); *n *= 1,986], adjusted for baseline age, sex, education, income, economic activity, neighborhood-level socioeconomic status, and traffic proximity (Model 1). dB(A), A-weighted decibels.

Associations between noise and depressive symptoms did not increase with increasing noise when exposure was categorized into four groups (Figure 2). When compared with the ≤ 55 dB(A) category, the association was strongest for the middle exposure category [> 60 to ≤ 65 dB(A), RR = 1.52; 95% CI: 1.11, 2.07] and equally weaker for the highest and lowest exposure groups (RR = 1.19; 95% CI: 0.85, 1.68 and RR = 1.19; 95% CI: 0.86, 1.65, respectively) (Figure 2). Similarly, there was no evidence of a monotonic dose–response relationship for nighttime road traffic noise, but the pattern differed: the middle exposure category [> 55 to ≤ 60 dB(A)] had the weakest association compared with the ≤ 50 dB(A) reference group (RR = 1.14; 95% CI: 0.78, 1.65) (see Supplemental Material, Figure S3).

Table 3 shows the results of additional analyses. We estimated a positive association between noise exposure and high depressive symptoms at follow-up among 2,115 participants with ≤ 13 years of education (Model 1 RR = 1.43; 95% CI: 1.10, 1.85), in contrast with a weak negative association among 1,185 participants with > 13 years of education (RR = 0.92; 95% CI: 0.56, 1.53). A higher effect estimate was found in the subgroup with insomnia at baseline (Model 1 RR = 1.62; 95% CI: 1.01, 2.59; *n* = 281) than in those without insomnia at baseline (RR 1.21; 95% CI: 0.94, 1.57; *n* = 2,803) (Table 3). The association between traffic noise and depressive symptoms did not change remarkably when excluding participants who reported to have/ever have had depression at baseline (*n* = 176) or had missing data on depression (*n* = 605), yielding an RR of 1.24 (95% CI: 0.97, 1.59; Model 1). Using a higher cutoff value for defining high noise exposure [L_den_ > 65 vs. ≤ 65 dB(A)] resulted in an RR of 1.07 (95% CI: 0.77, 1.49), which is in accord with the results shown in Figure 2. Using either only a CES-D score ≥ 17 (*n* = 244 cases at follow-up) or only intake of antidepressant medication (*n* = 157 cases at follow-up) to define the outcome did not produce results that were different from those obtained with the combined outcome definition (Table 3). In general, additional analyses for the association of nighttime traffic noise exposure > 50 dB(A) versus ≤ 50 dB(A) with high depressive symptoms at follow-up showed similar results to those for 24-hr noise exposure, with the possible exception of the analysis that used antidepressant medication use to define outcome (see Supplemental Material, Table S3).

**Table 3:** Results of the sensitivity analyses, showing relative risks (with 95% confidence intervals) of high depressive symptoms at follow-up in study participants exposed to residential road traffic noise (L_den_) > 50 dB(A) and ≤ 50 dB(A).

Subgroup	Cases (*n*)	Total (*n*)^*a*^	RR (95% CI)^*b*^
Education
≤ 13 years	214	1,968	1.43 (1.10, 1.85)
> 13 years	65	1,130	0.92 (0.56, 1.53)
Moved during follow-up
Yes	61	502	1.17 (0.72, 1.88)
No	218	2,596	1.33 (1.02, 1.72)
Insomnia
Yes	55	281	1.62 (1.01, 2.59)
No	222	2,803	1.21 (0.94, 1.57)
City of residence
Mülheim/Ruhr	99	1,162	1.21 (0.83, 1.76)
Bochum	89	927	1.51 (1.00, 2.29)
Essen	91	1,009	1.16 (0.77, 1.74)
Excluded lifetime prevalence of depression at baseline^*c*^	189	2,382	1.34 (1.01, 1.76)
Noise cutoff L_den_ > 65 dB(A)	279	3,098	1.07 (0.77, 1.49)
CES-D ≥ 17 only to define outcome	227	3,469	1.24 (0.96, 1.61)
Antidepressant medication only to define outcome	144	3,467	1.28 (0.92, 1.80)
Abbreviations: CES-D, Center for Epidemiologic Studies Depression Scale; CI, confidence interval; dB(A), A-weighted decibels; RR, relative risk. ^***a***^Maximum total *n* in Model 1 = 3,098; numbers differing from those in Table 1 reflect missing covariate data (in Model 1). ^***b***^Adjusted for age, sex, education (not in the education-stratified analysis), income, economic activity, neighborhood-level socioeconomic status, and traffic proximity (Model 1). No substantial differences were observed in unadjusted results and in results for Model 2 and Model 3 (data not shown). ^***c***^Excluded 176 participants who reported having/having ever had depression and 605 participants with missing data.			

## Discussion

Our prospective study provides support for the hypothesis that long-term exposure to road traffic noise may increase the risk of depressive symptoms.

In our study population as a whole, high depressive symptoms at follow-up were ~25–30% more frequent in study participants exposed to road traffic noise levels > 55 dB(A) than in participants exposed to noise levels ≤ 55 dB(A). The association remained stable after adjustment for various covariates, highlighting the robustness of the results when considering potential confounding factors. Our findings are in line with results from previous cross-sectional studies on road traffic noise and depression. A study conducted in Serbia (Stošić and Blagojević 2011) with 911 participants between 18 and 80 years old found that participants living in a noisy city area of Niš [daily period noise ≥ 55 dB(A) and night noise ≥ 45 dB(A)] reported “feeling depressed” more frequently than the control participants, who lived in two quiet city areas [daily period noise ≤ 55 dB(A) and night noise ≤ 45 dB(A)]. A similar small Swedish study compared 151 persons who lived in a quiet city area with 97 persons who lived in an area exposed to noise (Öhrström 1991). The study used mailed questionnaires to assess psychosocial wellbeing, including depression, and the authors found that people living in the noisy area felt depressed more often. In another questionnaire-based study of 366 women (20–60 years old) living in Tokyo (Yoshida et al. 1997), an unadjusted OR of 2.9 (*p* < 0.05) for high responses to depression-related questions was found for women exposed to residential road traffic noise levels > 70 dB(A) compared with those exposed to 45 to ≤ 70 dB(A). Importantly, none of these cross-sectional studies reported controlling for potential confounding factors. Sygna et al. (2014) found an association (controlled for confounders) between road traffic noise and psychological distress, including depressive symptoms, but only in a subgroup of 274 participants with low sleep quality (OR 1.40, 95% CI: 0.99, 1.98; per 10-dB increase). To our knowledge, the Caerphilly study (Stansfeld et al. 1996) is the only previous prospective study of traffic noise and depressive symptoms; in this study, the authors analyzed data from 1,725 men living in Caerphilly, South Wales (50–64 years old). This men-only study found no association between traffic noise levels at baseline [in four 5-dB(A) categories ranging from 51–55 dB(A) to 66–70 dB(A)] and mean depression scores from the general health questionnaire at the 5-year follow-up, adjusting for age, social class, noise sensitivity, and depressive symptoms at baseline (*n* = 1,587). However, the study did find an association with mean anxiety scores, which significantly differed across the noise categories (*p* for heterogeneity = 0.03, *n* = 1,584) (Stansfeld et al. 1996). In summary, most previous studies on road traffic noise and depressive symptoms found an association, and our study adds to the existing body of evidence by prospectively analyzing a comprehensive cohort including both men and women while at the same time accounting for potential confounding factors.

Sex-specific analyses revealed no differences between men and women. It is notable, however, that high depressive symptoms at follow-up were far more common in women than in men (12.7% vs. 5.9%). This result is consistent with existing epidemiologic research, where a higher prevalence of depression has been observed in women than in men, with an estimated female:male ratio of 2.3 (Wittchen et al. 2011). It has been argued that these differences in prevalence may not be real because depression symptoms may vary between men and women (Azorin et al. 2014; Rutz 1999; Schuch et al. 2014), but commonly applied diagnostic criteria focus on symptoms that are rather typical for women, and men are believed to display less pronounced help-seeking behavior than women (Piccinelli and Wilkinson 2000; Schuch et al. 2014). Thus, a potential for measurement error caused by sex-insensitive diagnostic criteria and varying prescribing patterns must be considered, and sex-specific associations deserve further attention.

When investigating different categories of road traffic noise, RRs did not increase linearly with increasing noise levels, and we found that elevated risks of high depressive symptoms were strongest not in the highest exposure group but in the intermediate exposure group for 24-hr noise exposure. However, the number of participants in the noise categories was small, the overall incidence of depressive symptoms was low, and we consider this analysis primarily exploratory for future research aims. Previous studies also failed to identify a linear trend (Stansfeld et al. 1996; Yoshida et al. 1997). An explanation for this missing dose–response relationship may be that measures for noise mitigation (e.g., noise protection windows) and behavioral prevention (i.e., closed windows, choice of quiet sleeping room, earplugs) may be more common in areas with very high noise exposure. A nonlinear relationship of exposure and outcome may also contribute to the inconsistency among the results from previous studies.

We found a strong association of traffic noise with high depressive symptoms in less-educated participants and a weak negative association in highly educated participants (Table 3). Furthermore, a high proportion of study participants with low incomes and low education and who were unemployed had high traffic-noise exposure (Table 1), supporting previous observations of a socially inequitable distribution of environmental burden (Braubach and Fairburn 2010). A previous analysis performed by the German Socio-Economic Panel found that low household income was associated with high perceived noise exposure (Kohlhuber et al. 2006).

The association of noise with depression-related outcomes that was observed in the HNR and in previous studies seems to be biologically plausible. Stratified analyses in the present study revealed a strong association between high noise exposure and high depressive symptoms in participants with insomnia at baseline, and the same was found in a previous study (Sygna et al. 2014). This finding is in line with the hypothesis of impaired sleep as a possible pathway for developing depressive symptoms (Baglioni et al. 2011). However, insomnia may also be a symptom of depression rather than a contributing factor; thus, an association between depression and insomnia at the same point in time may be bidirectional. Our results suggest that individuals with preexisting sleep disturbances might have increased vulnerability to the effects of noise on depressive symptoms. However, we do not know the underlying causes of insomnia in our study population.

Another factor linking noise and depression may be noise-induced stress reactions of the body. Acute noise stimuli cause the central nervous system to initiate warning/alert reflexes that are beyond individual control and that affect a number of bodily functions, such as muscle tension and pulse rate (Rylander 2004). Repeated exposure to noise for long periods is typically considered unpleasant or annoying when it interferes with activities of living such as communication, tasks that require concentration, or recreational activities such as sleep and rest. Habituation to noise rarely occurs, and chronic exposure to noise that causes negative physiological stress reactions may lead to a stage where acute effects, such as increased blood pressure, become permanent (Rylander 2004). Furthermore, it has been noted that exposure to stressors promotes neurochemical and endocrine changes that may be involved in the provocation of depressive disorder (Anisman and Merali 2002; Wager-Smith and Markou 2011). Chronic stress caused by noise exposure may lead to involuntary defeat reactions characterized by, for example, decreased motor function, reduced secretion of cortisol and adrenaline, and suppression of the immune system, with depression of mood a possible consequence. However, the extent to which noise causes such defeat reactions may differ among individuals depending on the ability to escape noise by, for example, closing the windows or choosing a bedroom facing away from the street (Rylander 2004). Increased stress hormone levels caused by noise are a frequent finding (Ising and Kruppa 2004) and may explain our observed results when we considered physiological stress as a factor in the pathway from noise exposure to depression. It is also possible that the observed association of noise with depressive symptoms is in part mediated by other stress-related or chronic diseases such as cardiovascular disease, which has been found to be associated with both noise and depression (Münzel et al. 2014; Hare et al. 2014); however, accounting for comorbidities by adjustment did not change the RR estimate in our study.

Strengths of this study include a high-quality noise exposure model and residential addresses obtained at baseline to accurately assess exposure. Depressive symptoms were assessed by a widely used and well-established instrument. The prospective design allowed investigation of long-term noise effects, assuming that the mean noise levels modeled for 2006 and assigned to the baseline (2000–2003) residence location were constant over the 5-year follow-up period. We were able to investigate a large number of randomly selected participants, allowing noise effects to be studied in different subgroups. Furthermore, comprehensive measurements enabled inclusion of many potential confounding factors in our analyses.

With regard to study limitations, exposure misclassification is a major concern in environmental epidemiology. Noise exposure assessment in the present study included residential road traffic noise only; other sources of residential noise, such as air or railway traffic noise or noise caused by neighbors, were not included. Nevertheless, road traffic is considered the major source of noise pollution in urban metropolitan contexts such as the investigated Ruhr area (Omidvari and Nouri 2009), and most of the neighborhoods included in our study population were not affected by aircraft noise. Furthermore, we had no information on time spent at the residence or on nonresidential noise exposures such as occupational noise. Individual characteristics such as room ventilation patterns, hearing ability, and noise protection windows were not accounted for in the analysis but may also have contributed to misclassification of noise exposure. Participants with (very) high levels of noise exposure may make more use of noise-avoidance strategies, which may lead to an underestimation of the effect that would be observed without these measures. This may in part explain our findings of a lower RR in the highest noise category. Participants exposed to high and low levels of noise may differ in some characteristics relevant to the development of depressive symptoms, and although we were able to take a range of these factors into account in our analyses, unknown confounding cannot be ruled out. Additional bias caused by missing data is possible; however, income information was the most commonly missing data, yet excluding those missing data from the crude model did not change the results. Potential air pollution effects were only accounted for indirectly by adjusting for traffic proximity. Modeling the average noise level, as we did here, does not reflect potential peaks, extreme noise events, or single sleep-disturbing noise events in otherwise quiet areas, all of which are of special relevance in terms of physiological stress reactions to noise (Rylander 2004; Babisch 2002). In addition, noise was modeled for the year 2006, and the assumption of unchanged noise exposure during the study period may not hold. The severity and presence of depressive symptoms vary over time; therefore, additional CES-D assessments (e.g., yearly instead of every 5 years) would have allowed for a more precise outcome measurement. We investigated a general population sample of middle-aged and older men and women living in a German metropolitan area; hence, our results cannot be generalized to populations from other countries, to children or young adults, or to populations residing in rural areas.

## Conclusion

Our results suggest that exposure to residential traffic noise may increase the risk of high depressive symptoms in middle-aged and older adults. Additionally, our study offers preliminary evidence that those with low socioeconomic status and those who experience sleep disturbances may be particularly vulnerable to noise effects. Further prospective research is needed to confirm the results of our study and to extend the generalizability of our findings to other populations. Studies including measures of stress and subjective noise annoyance may also extend our knowledge into the mechanisms of noise-induced depression. However, there is already evidence of adverse health effects arising from noise exposure, stressing the necessity of protecting populations from noise pollution; this is particularly important with regard to environmental justice because our results indicate that traffic noise may be unequally distributed across social strata.

## Supplemental Material

(425 KB) PDF
