# Supplementary material for: Residential Road Traffic Noise and High Depressive Symptoms after Five Years of Follow-up: Results from the Heinz Nixdorf Recall Study
Source: Environ Health Perspect. 2015 Nov 25;124(5):578–85. doi: 10.1289/ehp.1409400 (PMC4858388; doi:10.1289/ehp.1409400)
Supplement: (425 KB) PDF [file ehp.1409400.s001.acco.pdf]

Environ Health Perspect
DOI: 10.1289/ehp.1409400

Note to readers with disabilities: *EHP* strives to ensure that all journal content is accessible to all readers. However, some figures and Supplemental Material published in *EHP* articles may not conform to 508 standards due to the complexity of the information being presented. If you need assistance accessing journal content, please contact ehp508@niehs.nih.gov. Our staff will work with you to assess and meet your accessibility needs within 3 working days.

Supplemental Material

Residential Road Traffic Noise and High Depressive Symptoms after Five Years of Follow-up: Results from the Heinz Nixdorf Recall Study

Ester Orban, Kelsey McDonald, Robynne Sutcliffe, Barbara Hoffmann, Kateryna B. Fuks, Nico Dragano, Anja Viehmann, Raimund Erbel, Karl-Heinz Jöckel, Noreen Pundt, and Susanne Moebus

Table of Contents

Table S1. Characteristics of the Heinz Nixdorf Recall study participants that were excluded from our analysis.
Table S2. Relative risks (with 95% confidence intervals) of high depressive symptoms at follow-up in study participants exposed to residential nighttime road traffic noise (L_{night}) >50 dB(A) compared with ≤50 dB(A).
Table S3. Results of the sensitivity analyses, showing relative risks (with 95% confidence intervals) of high depressive symptoms at follow-up in study participants exposed to residential nighttime road traffic noise (L_{night}) >50 dB(A) compared with ≤50 dB(A).
Figure S1. Directed acyclic graph (DAG) on the hypothesized associations between road traffic noise, depressive symptoms and covariates in our study. Source: Created with DAGitty (www.dagitty.net, Textor et al. 2011).
Figure S2. Distribution of Heinz Nixdorf Recall study participants (n=3,300) by residential level of annual mean 24-hour (L_{den}) and nighttime noise (L_{night}) at the residential locations.
Figure S3. Relative risks and 95% confidence intervals of depressive symptoms at follow-up in association with exposure to different categories of nighttime noise compared with the

lowest noise category [≤50 dB(A); n=2,298], adjusted for baseline age, sex, education, income, economic activity, neighborhood-level socioeconomic status and traffic proximity (model 1).

Table S1. Characteristics of the Heinz Nixdorf Recall study participants that were excluded from our analysis.

Drop out 1 refers to those excluded due to missing information on depressive symptoms (CES-D, antidepressant medication use) or prevalent depressive symptoms (CES-D ≥17 and/or antidepressant medication use) at baseline. Drop out 2 refers to participants who died between baseline and follow-up, or had insufficient information on CES-D/antidepressant medication use to assess depressive symptoms at follow-up (see also table 1).

**Characteristics**	**Drop out 1 (N=1,025)**	**Drop out 2 (N=489)**
	N (%), mean ± SD, or median (Q1, Q3)	N (%), mean ± SD, or median (Q1, Q3)
**Baseline**		
Exposed to L_{den} >55 dB(A)	354 (34.5)	186 (38.0)
Exposed to L_{night} >50 dB(A)	243 (23.3)	138 (28.2)
Men	411 (40.1)	269 (55.0)
Age (years)	59.6 ± 8.0	62.2 ± 8.3
CES-D ≥17 and/or antidepressant medication	593 (100.0)	0 (0.0)
N missing	432	0
CES-D ≥17	449 (52.5)	0 (0.0)
N missing	169	0
Antidepressant medication	221 (31.1)	0 (0.0)
N missing	314	0
Insomnia	220 (21.8)	54 (11.3)
N missing	17	10
Number of co-morbidities[a]		
0	366 (35.7)	155 (31.7)
1	295 (28.8)	155 (31.7)
≥ 2	364 (35.5)	179 (36.6)
Reported lifetime prevalence of depression	213 (24.5)	36 (8.9)
N missing	157	86
Body mass index	28.2 ± 4.8	28.4 ± 4.6
N missing	17	2
Smoking		
current	268 (26.3)	149 (30.7)
former	304 (29.9)	161 (33.1)
never	446 (43.8)	176 (36.2)
N missing	7	3

(Table S1 continued)

Distance to nearest major road (meters)	811.6 (428.1, 1400.1)	859.2 (407.2, 1507.0)
N missing	3	1
Unemployed in neighborhood (%)	13.0 ± 3.7	12.9 ± 3.5
Education[b]		
≤ 10 years	194 (18.9)	92 (18.8)
11–13 years	576 (56.2)	262 (53.6)
14–17 years	181 (17.7)	111 (22.7)
≥ 18 years	74 (7.2)	24 (4.9)
N missing	0	1
Household net income		
Quartile 1 (low)	318 (33.8)	149 (32.5)
Quartile 2	217 (23.1)	111 (24.2)
Quartile 3	229 (24.3)	107 (23.3)
Quartile 4 (high)	177 (18.8)	92 (20.0)
N missing	84	30
Economic activity		
employed	354 (34.9)	132 (27.6)
inactive	567 (55.9)	330 (67.9)
unemployed	93 (9.2)	22 (4.5)
N missing	11	3
City of residence		
Mülheim	355 (34.7)	163 (33.3)
Bochum	270 (26.4)	142 (29.0)
Essen	399 (39.0)	184 (37.6)
**Follow-up**		
CES-D ≥17 and/or antidepressant medication	308 (36.8)	0 (0.0)
N missing	188	489
CES-D ≥17	226 (26.8)	0 (0.0)
N missing	180	441
Antidepressant medication	140 (16.7)	0 (0.0)
N missing	186	466

Q1 and Q3= quartile 1 (25th percentile) and quartile 3 (75th percentile)

[a] Of the following: myocardial infarction, heart failure, stroke, diabetes, emphysema, asthma, cancer, rheumatism, slipped disc, migraine

[b] Combining school and vocational training

Table S2. Relative risks (with 95% confidence intervals) of high depressive symptoms at follow-up in study participants exposed to residential nighttime road traffic noise (L_{night}) >50 dB(A) compared with ≤50 dB(A).

Model	N cases	N total	RR (95% CI)
**Unadjusted**			
total	302	3,300	1.30 (1.03, 1.64)
men	101	1,715	1.31 (0.87, 1.97)
women	201	1,585	1.31 (0.99, 1.73)
**Model 1**[a]			
total	279	3,098	1.29 (1.01, 1.64)
men	98	1,650	1.19 (0.77, 1.82)
women	181	1,448	1.36 (1.01, 1.82)
**Model 2**[b]			
total	278	3,089	1.30 (1.02, 1.65)
men	98	1,644	1.19 (0.76, 1.86)
women	180	1,445	1.37 (1.02, 1.83)
**Model 3**[c]			
total	276	3,075	1.29 (1.01, 1.64)
men	97	1,637	1.14 (0.74, 1.76)
women	179	1,438	1.39 (1.03, 1.86)

[a] Adjusted for age, sex (except in the sex-stratified analysis), education, income, economic activity, neighborhood-level socioeconomic status, traffic proximity
[b] Additionally adjusted for body mass index, smoking
[c] Additionally adjusted for co-morbidities, insomnia

Table S3. Results of the sensitivity analyses, showing relative risks (with 95% confidence intervals) of high depressive symptoms at follow-up in study participants exposed to residential nighttime road traffic noise (L_{night}) >50 dB(A) compared with ≤50 dB(A).

**Subgroup**	**N cases**	**N total**[a]	**RR (95% CI)**[b]
Education			
≤13 years	214	1,968	1.38 (1.05, 1.81)
>13 years	65	1,130	1.01 (0.57, 1.79)
Moved during follow up			
yes	61	502	1.34 (0.82, 2.20)
no	218	2,596	1.27 (0.96, 1,68)
Insomnia			
yes	55	281	1.56 (0.96, 2.52)
no	222	2,803	1.24 (0.94, 1.63)
City of residence			
Mülheim/R	99	1,162	1.26 (0.85, 1,87)
Bochum	89	927	1.36 (0.88, 2.11)
Essen	91	1,009	1.23 (0.79, 1.90)
Excluded lifetime prevalence of depression at baseline[c]	189	2,382	1.31 (0.98, 1.76)
Noise cutoff L_{night} >60 dB(A)	279	3,098	1.20 (0.75, 1.93)
CES-D ≥17 only to define outcome	227	3,469	1.31 (1.00, 1.71)
Antidepressant medication only to define outcome	144	3,467	1.07 (0.74, 1.55)

[a] max. total N in model 1=3,098, numbers differing from those in table 1 reflect missing covariate data (in model 1)

[b] Adjusted for age, sex, education (not in the education-stratified analysis), income, economic activity, neighborhood-level socioeconomic status and traffic proximity (model 1); no substantial differences were in unadjusted and model 2 and 3 results (data not shown)

[c] Excluded 176 who reported having/having ever had depression and 605with missing data

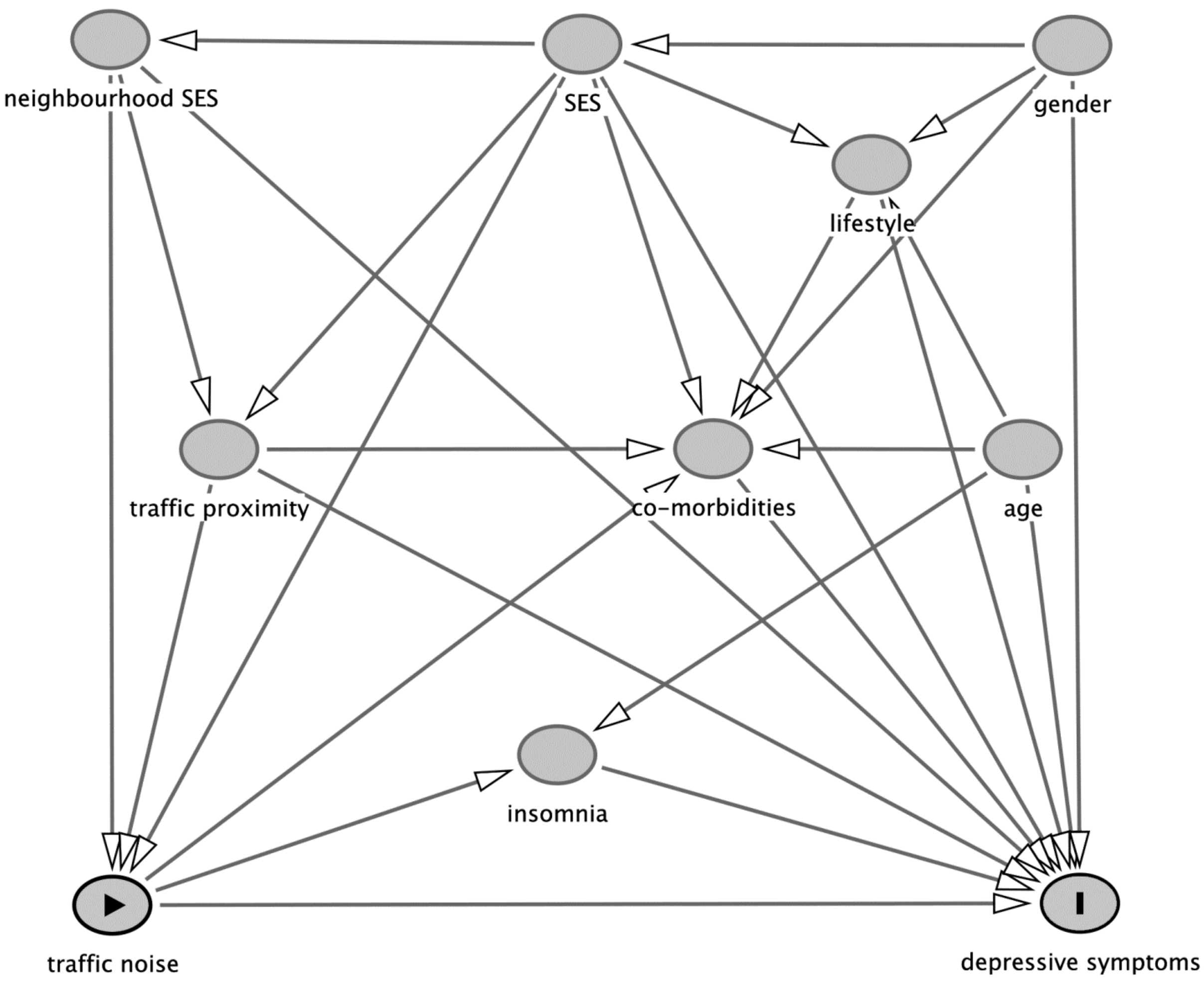

Figure S1. Directed acyclic graph (DAG) on the hypothesized associations between road traffic noise, depressive symptoms and covariates in our study. Source: Created with DAGitty (www.dagitty.net, Textor et al. 2011)

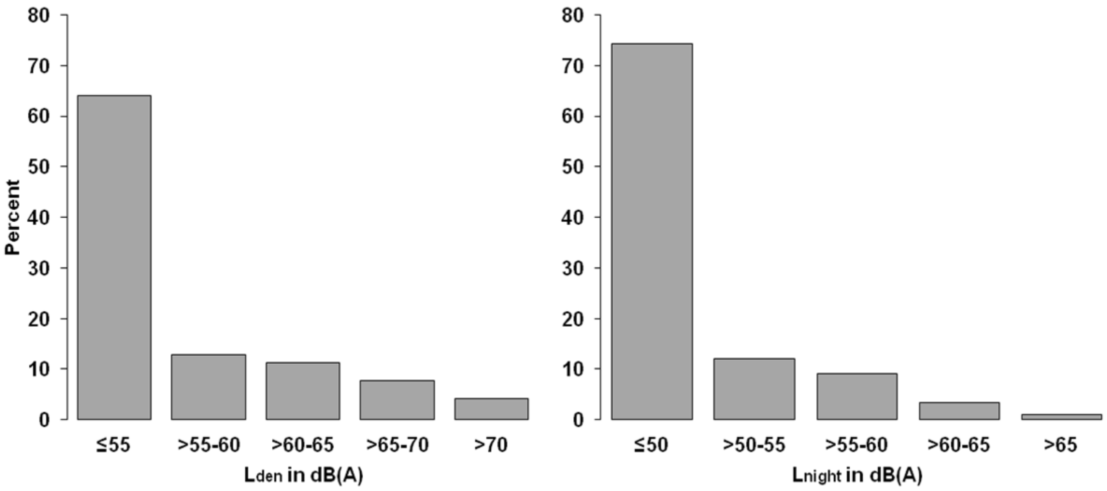

Figure S2. Distribution of Heinz Nixdorf Recall study participants (n=3,300) by residential level of annual mean 24-hour (L_{den}) and nighttime noise (L_{night}) at the residential locations.

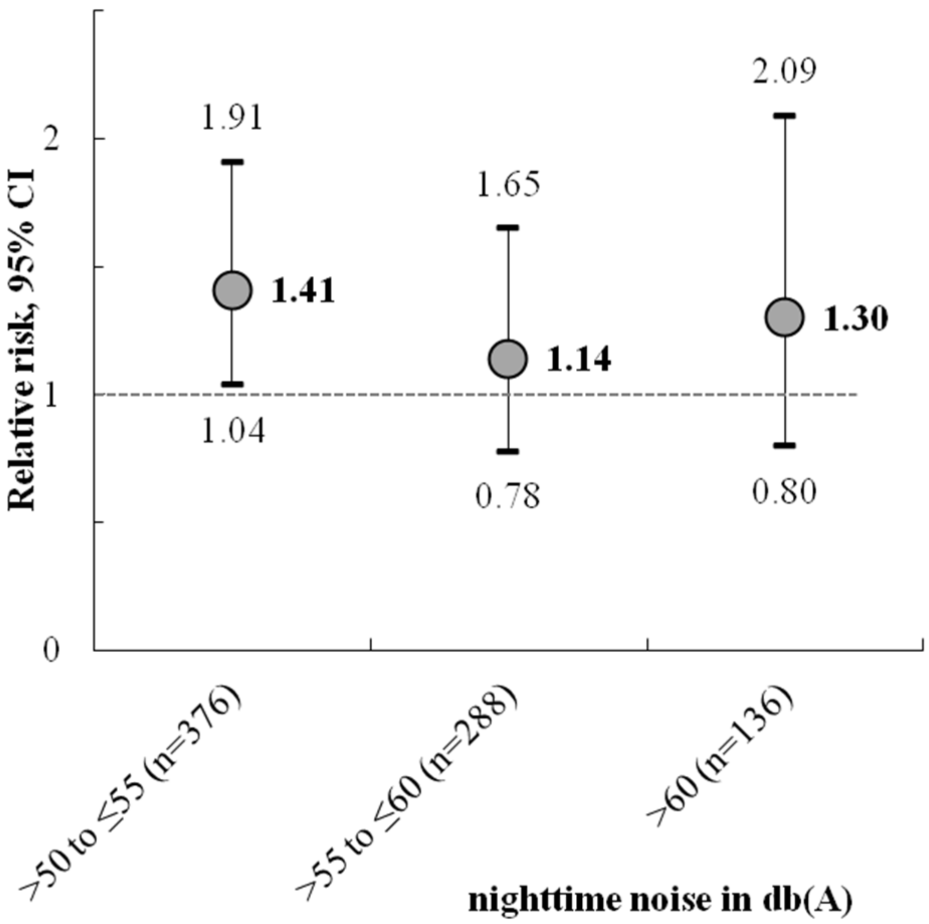

Figure S3. Relative risks and 95% confidence intervals of depressive symptoms at follow-up in association with exposure to different categories of nighttime noise compared with the lowest noise category [≤50 dB(A); n=2,298], adjusted for baseline age, sex, education, income, economic activity, neighborhood-level socioeconomic status and traffic proximity (model 1).
